# Editorial: Brain serotonergic system

**DOI:** 10.3389/fnsyn.2023.1225731

**Published:** 2023-06-07

**Authors:** Gary C. Mouradian, Matthew A. Cooper

**Affiliations:** ^1^Neuroscience Research Center, Cardiovascular Research Center, Department of Physiology, Medical College of Wisconsin, Milwaukee, WI, United States; ^2^NeuroNET Research Center, Department of Psychology, University of Tennessee Knoxville, Knoxville, TN, United States

**Keywords:** brain serotonin, 5-HT, serotonin, CNS, CNS serotonin, neuromodulation, serotonergic modulation

The CNS serotonin (5-HT) system is an impressive and diverse neurochemical system. With billions of neurons in the human brain, only about half a million are serotonergic (Hornung, 2003) and reside within the brainstem. And yet, through widespread projections throughout the brain, virtually all human behaviors and biological functions involve the serotonergic system. Despite the well-recognized importance of 5-HT in biology, many outstanding questions remain. The goal of this Research Topic was to extend our understanding of the importance and role of the 5-HT system during development, mechanisms contributing to 5-HT neuron diversity, interaction with other neural or hormonal systems, and influence on behavior and pain perception.

Brainstem serotonin neurons are important for proper neural control of breathing. Critical to regulating breathing, from birth to death, is the ability to continuously monitor CO_2_ and pH in the body. Subpopulations of brainstem 5-HT neurons participate in this function and their dysfunction is implicated in Sudden Infant Death Syndrome, commonly known as SIDS (Duncan et al., 2010). While chemosensitive 5-HT neurons are identified by developmental expression of Pet1 and Egr2 development (Brust et al., 2014), the underlying mechanisms of CO_2_ or pH sensing by these neurons are unknown and post-natal molecular and/or functional markers differentiating between CO_2_/pH sensitive vs. insensitive 5-HT neurons are non-existent. Using a novel and cutting-edge approach coupling electrophysiology with subsequent single cell RNA Sequencing, abbreviated as Patch-to-Seq, Mouradian et al. used multiple unbiased and biased bioinformatic analyses to highlight the potential role of two unexpected candidate genes, CD46 and Iba57, as at least gene markers of CO_2_/pH sensitive neurons. Moreover, the authors provide robust documentation of firing patterns that provide high predictability in differentiating between 5-HT CO_2_/pH sensitive and insensitive neurons. These data provide novel insights into transcriptional control of an important autonomic function and highlight the utility of a unique method to further link and dissect 5-HT function and gene expression.

Stress-sensitive 5-HT neurons are located in the caudal portions of the dorsal raphe nucleus (DRN), and these 5-HT neurons send robust projections to the limbic system where they activate multiple types of 5-HT receptors (Lowry et al., 2008). While voluntary exercise creates plasticity in the 5-HT system, improves mood, and reduces fear and anxiety, the role of 5-HT2 receptors in the bed nucleus of the stria terminalis (BNST) is unknown. Fox et al. addressed this gap in the literature by using a mouse model to test whether voluntary exercise leads to reduced 5-HT2C receptor function in the BNST. They used the anxiogenic drug meta-chlorophenylpiperazine (mCPP) with high affinity for 5-HT2B and 5-HT2C receptors and found that voluntary wheel running prevents the dose-dependent increase in acoustic startle response produced by mCPP infusion in the BNST. While mCPP infusion into the dorsal hippocampus or basolateral and central complex of the amygdala did not alter acoustic startle, excitotoxic lesions of the BNST prevented the effects of systemic mCPP treatment. Lastly, RNAscope showed that voluntary exercise reduces 5-HT2C transcripts in the BNST. Altogether, these findings suggest that downregulation of 5-HT2C receptors in the BNST may be a key mechanism by which voluntary exercise attenuates fear and anxiety. Further, these results contribute to a growing body of literature that neural plasticity in the 5-HT system is essential for experience-dependent changes in stress resilience.

While glutamatergic and GABAergic signaling are known to modulate pain and anxiety, less is known about the influence of the serotonergic system. In their review, Hao et al. outline the recent advances that have been made identifying how the 5-HT system modulates chronic pain and injury-related anxiety. They highlight central pain- and anxiety-related pathways modulated by serotonergic inputs which is determined by the type of serotonin receptor(s) expressed. The serotonergic system can also contribute to injury-related anxiety, based on recent results (Zhou et al., 2022) showing that greater 5-HT in the anterior cingulate cortex could attenuate pain-induced LTP in a 5-HT receptor-specific manner. Relevant to anxiety, the insular cortex and amygdala can be modulated by 5-HT receptors to promote or reduce anxiety-like behaviors. The effects on chronic pain and pain-related anxiety can be mediated at the synapse, within the relevant pain-processing brain regions, through serotonergic modulation of LTP and LTD mechanisms. Together with Fox et al., these studies provide a summary for how and why targeting the serotonergic system to improve chronic pain and pain-related anxiety is a promising area of future study.

The final publication of this Research Topic from Voronezhskaya highlights the utility of simplified organ systems and a reminder of the idea, provided by Sakharov (1990), that 5-HT is a general modulator or integrating molecule at the level of the whole-organism. Specifically, she provides a summary of her views and idea about how 5-HT is a volume transmission signal (as opposed to wired-transmission) during development and as an integrative factor underlying coordinated behaviors in the mollusk. Bioactive compounds, like 5-HT, are involved in controlling developmental behaviors, like neurite outgrowth. Indeed, some of the earliest cells of the apical sensory organ (ASO)—termed the “larval brain”—of mollusks express 5HT. This region is an active source for 5-HT as other portions of the mollusk develops, providing a 5-HT gradient which is critical for correct growth of pioneer axons and formation of ganglia. Such cells of the ASO are also a means for neighboring adult mollusks to communicate with offspring pertaining to overcrowding or starvation, which triggers adaptive developmental behaviors for the offspring to follow to maximize survival. Together, evidence is provided to support the argument that 5-HT is a critical volume transmission molecule.

Manuscripts in this Research Topic highlight the diverse and impressive involvement, function, and effects of the brain serotonin system. These studies indicate that heterogenous populations of 5-HT neurons in the brain and diverse sets of receptors enable the 5-HT system to modulate an enormous array of behavioral, emotional, cognitive, and behavioral functions from the onset of development. Indeed, the brain 5-HT system has important contributions critical to life at the subconscious level, but also in modulating how we interact and sense the external world.

## Author contributions

GM and MC contributed to writing, editing, and finalizing the text in this editorial. GM initiated the editorial. All authors contributed to the article and approved the submitted version.
